# Ability of high fat diet to induce liver pathology correlates with the level of linoleic acid and Vitamin E in the diet

**DOI:** 10.1371/journal.pone.0286726

**Published:** 2023-06-02

**Authors:** Dalton S. Graham, Gang Liu, Ailar Arasteh, Xiao-Ming Yin, Shengmin Yan

**Affiliations:** Department of Pathology and Laboratory Medicine, Tulane University School of Medicine, New Orleans, Louisiana, United States of America; Tokyo University of Agriculture, JAPAN

## Abstract

Increased uptake of fat, such as through the ingestion of high fat diet (HFD), can lead to fatty liver diseases and metabolic syndrome. It is not clear whether certain fatty acids may be more pathogenic than others to the liver. Linoleic acid (LA) is the most abundant polyunsaturated fatty acid in the Western diet and its excessive consumption can lead to increased lipid peroxidation. We hypothesized that a high level of LA in HFD will contribute significantly to the hepatic steatosis and injury, whereas vitamin E (VIT-E) may reverse the effects from LA by inhibiting lipid peroxidation. To test this hypothesis, we fed mice with the following diets for 20 weeks: a standard low-fat diet (CHOW), HFD with a low level of LA (LOW-LA, 1% of energy from LA), HFD with a high level of LA (HI-LA, 8% of energy from LA), or HI-LA diet with VIT-E supplement (HI-LA + VIT-E). We found that the HI-LA diet resulted in more body weight gain, larger adipocyte area, and higher serum levels of triglycerides (TG) and free fatty acids (FFA) relative to the CHOW and LOW-LA diets. In mice fed with the HI-LA diet, severer hepatic steatosis was seen with higher levels of hepatic TG and FFA. Expression of genes related to lipid metabolism was altered in the liver by HI-LA diet, including fibroblast growth factor 21 (*Fgf21*), cluster of differentiation 36 (*Cd36*), stearoyl-CoA desaturase 1 (*Scd1*), and acyl-CoA oxidase 1 (*Acox1*). Liver injury, inflammation and fibrotic response were all enhanced in mice fed with the HI-LA diet when compared with the LOW-LA diet. Notably, addition of VIT-E supplement, which restores the proper VIT-E/PUFA ratio, significantly reduced the detrimental effects of the high level of LA. Taken together, our results suggest that a high level of LA and a low ratio of VIT-E/PUFA in HFD can contribute significantly to metabolic abnormalities and hepatic injury.

## Introduction

Nonalcoholic fatty liver disease (NAFLD) has become a global epidemic, with an estimated 25% worldwide prevalence [1]. Nearly one-third of cases will progress from simple steatosis to nonalcoholic steatohepatitis (NASH) and fibrosis, which is associated with a significantly higher mortality [1–3]. NASH was first recognized in 1980 [4], but its prevalence has only grown since then. Diet has been long recognized to contribute to the development of NAFLD, but the specific components of concern are debated. Thus, recognizing the changes in intake of dietary components during the modern era should provide plausible hypotheses of potential culprits.

The greatest change of per capita food consumption over the 20th century in the United States was the increase in intake of cooking oils, an over 2000 percent increase [5]. Consumption in oils rich in linoleic acid (LA), an omega-6 polyunsaturated fatty acid (PUFA), including soybean, canola, peanut, corn, and safflower oils, all increased significantly. This amounted to an increase in the percentage of total energy consumed as LA from 2.79% in 1909 to 7.21% in 1999, despite the intake of total fat remaining constant. Given that consumption of these oils continues to increase over the past two decades, LA consumption is likely well over the estimated percentage in 1999 [6]. LA is the most abundant polyunsaturated fatty acid in the US diet and its excessive consumption may cause consequences in human health [7].

PUFAs are distinct in their biological activity from monounsaturated fatty acids (MUFA) and saturated fatty acids (SFA). PUFAs are susceptible to non-enzymatic chemical oxidation (lipid peroxidation), whereas MUFA and SFA are not [8]. They can also undergo enzymatic conversion into a variety of pro-inflammatory mediators including prostaglandins and leukotrienes. Peroxided lipids are implicated in human diseases, including fatty liver diseases [9–11], while vitamin E (VIT-E) has been shown to protect against non-enzymatic lipid peroxidation [12]. High LA intakes correlate with increased concentrations of peroxided bioactive metabolites, while reducing LA can decrease them [13]. The increase in prevalence of nonalcoholic liver diseases over the same time period as the increase in LA consumption warrants investigation into the possible effect of LA on the liver and systemic metabolism. However direct evidence suggesting the role of LA in the development of fatty liver diseases was lacking. It is also unclear whether VIT-E can improve fatty liver diseases by reducing lipid peroxidation.

Current animal models for NAFLD may use diets that contain high fat, high fructose, and/or high cholesterol, or that are deficient in methionine and choline [14]. However, these diets deserve scrutiny for their lack of similarity to a feasible human diet. There was increased adiposity and metabolic abnormalities in animals given diets containing high amounts of LA independent of the fat level, while low LA-containing diets did not have this effect [15, 16]. However, pathological changes in the liver were not further explored. Here, we hypothesize that the amount of LA is a significant contributing factor for liver pathogenesis in the HFD model of mouse NAFLD, and VIT-E can play a protective role in LA-induced pathological changes.

## Materials and methods

### Animals

Male C57BL/6 mice at the age of 10 weeks were housed in specific pathogen fee environment with a 12:12 light-dark cycle. Male mice were chosen because female mice are usually resistant to diet-induced obesity [17]. Mice were randomly divided into 4 groups (4 mice per group) and fed with indicated diet for 20 weeks. Animals were then euthanized by exsanguination under anesthetic (250 mg/kg body weight Tribromoethanol via intraperitoneal injection) to harvest tissues of interest in the afternoon without fasting. No adverse events were observed in animals. All studies were conducted per the NIH guideline and approved by the IACUC of Tulane University.

### Diets

The standard low fat chow diet (PicoLab Rodent Diet 20/LabDiet 5053) was provided by the facility and sourced from Lab Diet (St. Louis, MO). High-fat diets (with 35% kcals from fat) with low or high levels of LA, or with a high level of LA plus VIT-E supplements (DYETS #104946, #104947, and #181162, respectively) were customarily prepared by Dyets, Inc. (Bethlehem, PA). Nutrients compositions in terms of percentage calorie contribution, amount of VIT-E in diet and the ratio of VIT-E vs PUFA (Table 1) are calculated using data provided by the manufacturers or obtained from USDA’s food data central (https://fdc.nal.usda.gov/) as in S1 and S2 Tables. The calorie contribution of LA to the HFD is preset through customarily manufacture at 1% for Low-LA diet and 8% for High-LA diet. Commonly used high-fat diet (e.g. Research diets D12492, with 60% kcals from fat) contains around 15% LA.

**Table 1:** Percentage calorie contribution of different nutrients in the four diets.

	CHOW	LOW-LA	HI-LA	HI-LA + VIT-E
**Protein**	23.3	18.4	18.4	18.4
**Carbohydrate**	64.2	46.2	46.3	46.3
**Fat**	12.5	35.4	35.3	35.3
SFA	2.2	21.7	15.1	15.1
MUFA	2.8	11.5	11.0	11.0
LA	6.5	1.0	8.0	8.0
Other PUFA	1.0	1.2	1.2	1.2
**VIT-E/Diet (mg/kg)**	44.6	20.7	32.4	78.0
**VIT-E/PUFA (mg/g)**	1.7	2.2	0.8	1.9

1. The calorie contribution of protein, carbohydrate and fat is calculated using diet composition data provided by the manufacturers.

2. The calorie contribution of different fatty acids in the fat is calculated in S1 Table.

3. The level of VIT-E in the diet and the ratio vs that of PUFA are calculated in S2 Table.

Abbreviation: LOW-LA, HFD with 1% calories contribution from linoleic acid; HI-LA: HFD with 8% calories contribution from linoleic acid; HI-LA + VIT-E, HFD with 8% calories contribution from linoleic acid and supplemented with VIT-E.

### Biochemistry analysis

Serum alanine aminotransferase (ALT), a marker for liver damage, was measured using a kit provided by Pointe Scientific (Canton, MI, Cat no. 23-666-087). Liver malondialdehyde (MDA), a marker for lipid peroxidation, was determined using a kit by Biovision (Exton, PA, Cat no. K454). Free fatty acids in serum and liver were measured by Biovision’s assay (Free Fatty Acid Quantification Colorimetric/Fluorometric Kit, Cat no. K612). Triglycerides (TG) and total cholesterol (TC) were quantified in both serum and liver, using kits provided by Pointe Scientific (Canton, MI, Cat no. 23-666-411 and C7510-120) as previously described [18].

### Histology

Samples of the right liver lobe and the inguinal white adipose tissue (iWAT), a subcutaneous adipose region, and the epididymal white adipose tissue (eWAT), a visceral adipose region, were dissected. Tissues were fixed in 10% formalin for 48–72 hours prior to hematoxylin and eosin (H&E) or trichrome staining, or immunostaining with anti-F4/80. Images were taken using an Olympus BX60 microscope equipped with Olympus DP71 camera. At least 5 fields at the indicated magnification were taken per mouse sample. The number of F4/80+ macrophages was manually quantified in 200x fields. Adipocyte size (in area) was quantified in 200x fields with ImageJ, and the size frequency distribution is calculated as previously described [19].

### qRT-PCR

RNA was isolated from livers using GeneJET RNA Purification Kit (Thermo Fisher, Waltham, MA, Cat no. K0731). The cDNA was prepared using M-MLV Reverse Transcriptase (Thermo Fisher kit, Waltham, MA, Cat no. 28025013). Quantitative real time polymerase chain reaction (qRT-PCR) was performed using PowerTrack™ SYBR Green Master Mix (Thermo Fisher kit, Waltham, MA, Cat no. A46113) with a Quant Studio 3 Real-Time PCR System. Fold change of expression was calculated with the 2^-ΔΔCt^ method [18]. The level of β-actin was chosen as the internal control for normalization. PCR primers used in this study are listed in Table 2.

**Table 2:** Primer list.

Gene name	Sequence (Forward)	Sequence (Reverse)
***Acaca***	5’-GCCTCTTCCTGACAAACGAG-3’	5’-TGACTGCCGAAACATCTCTG-3’
***Acadl***	5’-GGTGGAAAACGGAATGAAAGG-3’	5’-GGCAATCGGACATCTTCAAAG-3’
***Acadm***	5’-TGTTAATCGGTGAAGGAGCAG-3’	5’-CTATCCAGGGCATACTTCGTG-3’
***Acox1***	5’-CATATGACCCCAAGACCCAAG-3’	5’-CATGTAACCCGTAGCACTCC-3’
***Actin***	5’-ACTATTGGCAACGAGCGGTT-3’	5’-CAGGATTCCATACCCAAGAAGGA-3’
***Apob***	5’-ATTCGAGCACAGATGACCAG-3’	5’-GTACCTTTCACCATCAGACTCC-3’
***Apoe***	5’-CAATTGCGAAGATGAAGGCTC-3’	5’-TAATCCCAGAAGCGGTTCAG-3’
***Cd36***	5’-GCGACATGATTAATGGCACAG-3’	5’-GATCCGAACACAGCGTAGATAG-3’
***Fabp1***	5’-TCTCCGGCAAGTACCAATTG-3’	5’-TTGATGTCCTTCCCTTTCTGG-3’
***Fasn***	5’-CCCTTGATGAAGAGGGATCA-3’	5’-ACTCCACAGGTGGGAACAAG-3’
***Fgf21***	5’-TACACAGATGACGACCAAGA-3’	5’-GGCTTCAGACTGGTACACAT-3’
***Lipa***	5’-AAGGTCCCAGACCAGTTGTG-3’	5’-TGTGCTTCAGAGACCAGGTG-3’
***Scd1***	5’-TTCTTACACGACCACCACCA-3’	5’-CCGAAGAGGCAGGTGTAGAG-3’
***Srebf1***	5’-CAAGGCCATCGACTACATCCG-3’	5’-GCCCTCCATAGACACATCTGT-3’

### Statistical analysis

Statistical analyses were performed using GraphPad Prism 9. Student *t*-test was used to determine differences between 2 groups. Differences among more than 2 treatment groups were determined by one-way analysis of variance (ANOVA) followed by *Turkey* post hoc test. Results were considered statistically significant for P values < 0.05.

## Results

### A high linoleic acid diet increases body weight without elevating energy intake

**Fig 1:**
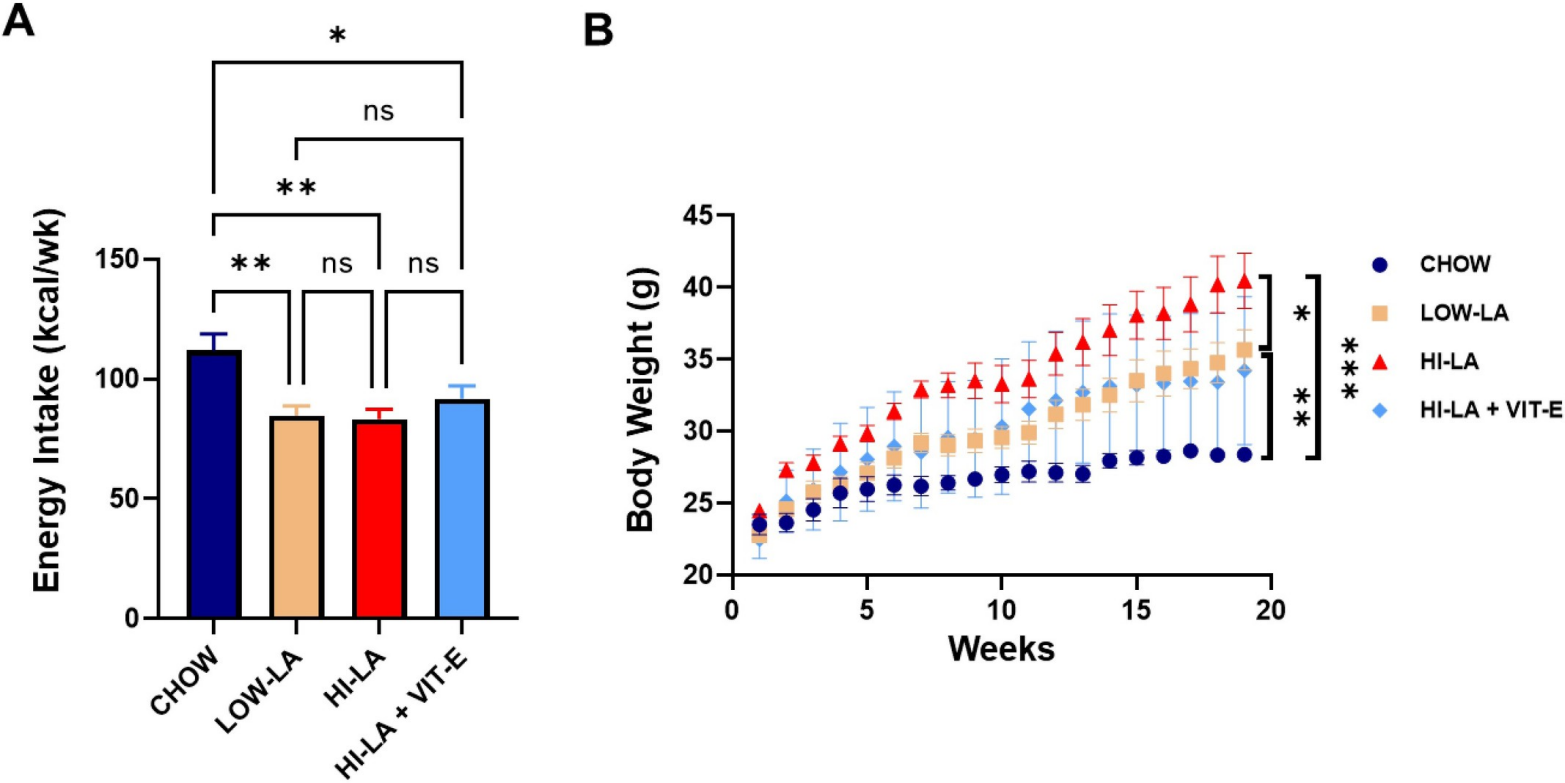
Effects of linoleic acid on food consumption and body weight. Male mice were fed with one of the four types of diets for 20 weeks. (A). Weekly average energy intake was calculated based on the amount of food consumed by each group of mice and the energy density values of the diet provided by the manufacturer. (B). Body weight of each group of mice measured weekly. Data were presented as means ± S.E., *p<0.*05*, **p<0.*01*, ***p<0.*001*, ns, no significance.

Mice were fed with the standard control diet (CHOW), the low linoleic acid diet (LOW-LA), high linoleic acid diet (HI-LA), or high linoleic acid diet supplemented with VIT-E (HI-LA + VIT-E). VIT-E was added to high LA diet to maintain a VIT-E/PUFA ratio (1.9) that is similar to those in the chow diet (1.7) and LOW-LA diet (2.2) (Table 1). Mice fed with the CHOW diet had the highest weekly food consumption of any group (Fig 1A). Food consumption did not significantly differ among the three HFD groups. The CHOW-fed mice had the lowest final body weight, which was significantly lower than the LOW-LA and HI-LA + VIT-E fed groups (Fig 1B). The HI-LA fed mice exhibited the highest final body weight, which was significantly greater than the CHOW and LOW-LA fed groups, even though the groups did not differ statistically in energy intake, indicating a higher obesogenic effect of the HI-LA diet (Fig 1B). Notably, the extra weight gain in the HI-LA group was significantly counteracted by VIT-E supplementation so that the average body weight of the mice fed the HI-LA + VIT-E diet was about the same as that of the LOW-LA fed group.

### A high linoleic acid diet induces adipocyte hypertrophy

**Fig 2:**
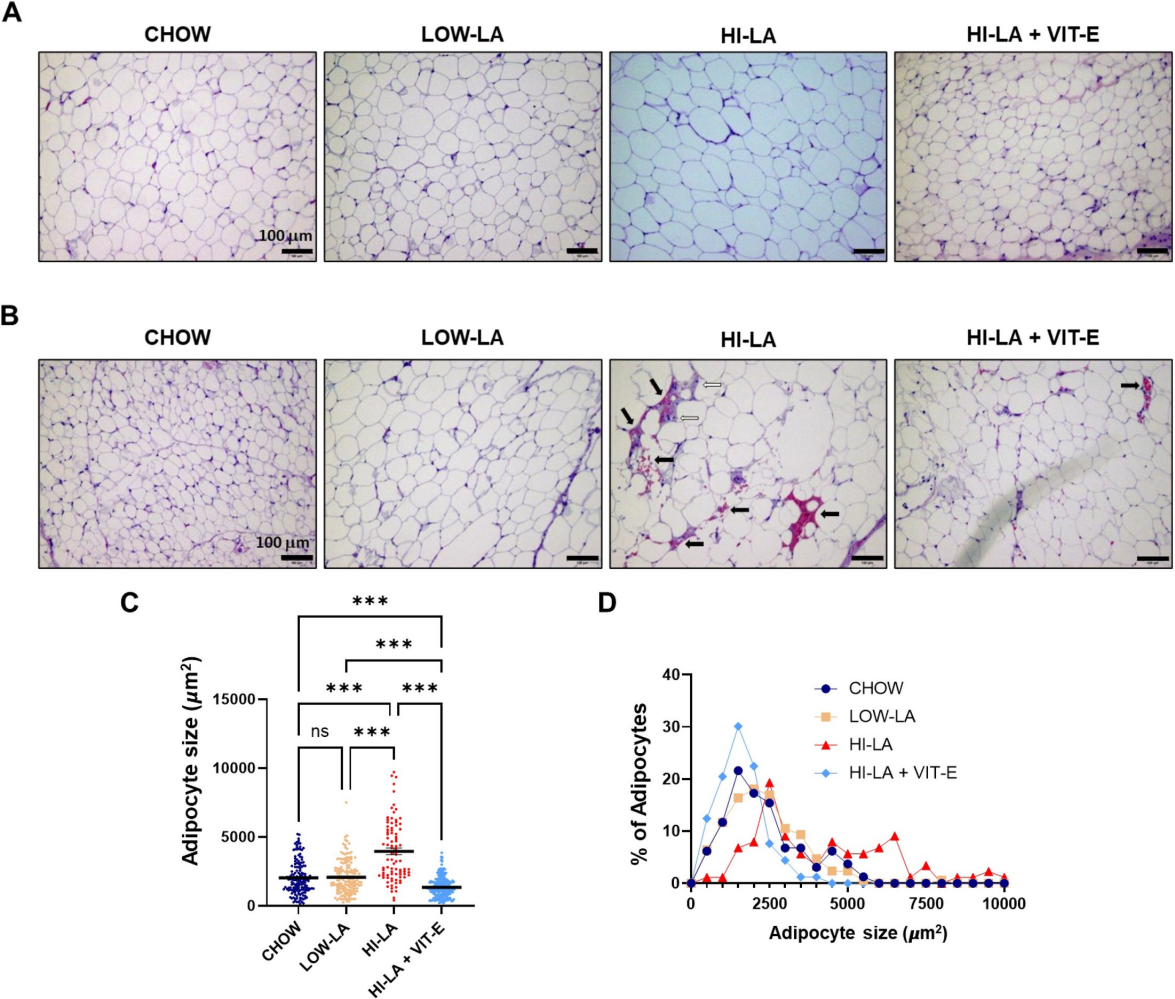
Effects of linoleic acid on the size of adipose tissues. (A-B). Representative H&E images of iWATs (A) and eWATs (B). White arrows indicate inflammatory cells and black arrows indicate vessel-like structures and red blood cells (B). Scale bar: 100 μm. (C-D). Adipocyte size (C) and size distribution (D) in iWATs were determined (n = 162, 171, 88 and 249 for the four groups, respectively). The horizontal bar represents the mean value of the size (C). Data were presented as means ± S.E., ***p<0.*001*, ns, no significance.

Histological examination showed little evidence of inflammation in any of the iWAT samples analyzed (Fig 2A). Interestingly, notable presence of inflammatory cells were found in sections of epididymal white adipose tissue (eWAT) of the mice fed with the HI-LA diet (Fig 2B). There were also higher amounts of red blood cells in eWAT, suggesting increased angiogenesis [20]. It is not clear why there is such a difference in the level of inflammation or angiogenesis between iWAT and eWAT.

To correlate with the body weight and other systemic metabolic changes, we quantified the size of the adipocytes in iWAT. The average adipocyte size was significantly greater in mice fed the HI-LA diet than those in other groups (Fig 2C). Thus, the HI-LA diet produced a greater proportion of adipocytes at larger sizes than any of the other groups (Fig 2D). Consistent with the reduced body weight gain, the HI-LA + VIT-E fed mice had similar adipocyte size to that seen in the CHOW and LI-LA group (Fig 2C and 2D). The increased size of adipocytes could explain the increased body weight of the mice in the HI-LA group.

### A high linoleic acid diet alters lipid metabolism

**Fig 3:**
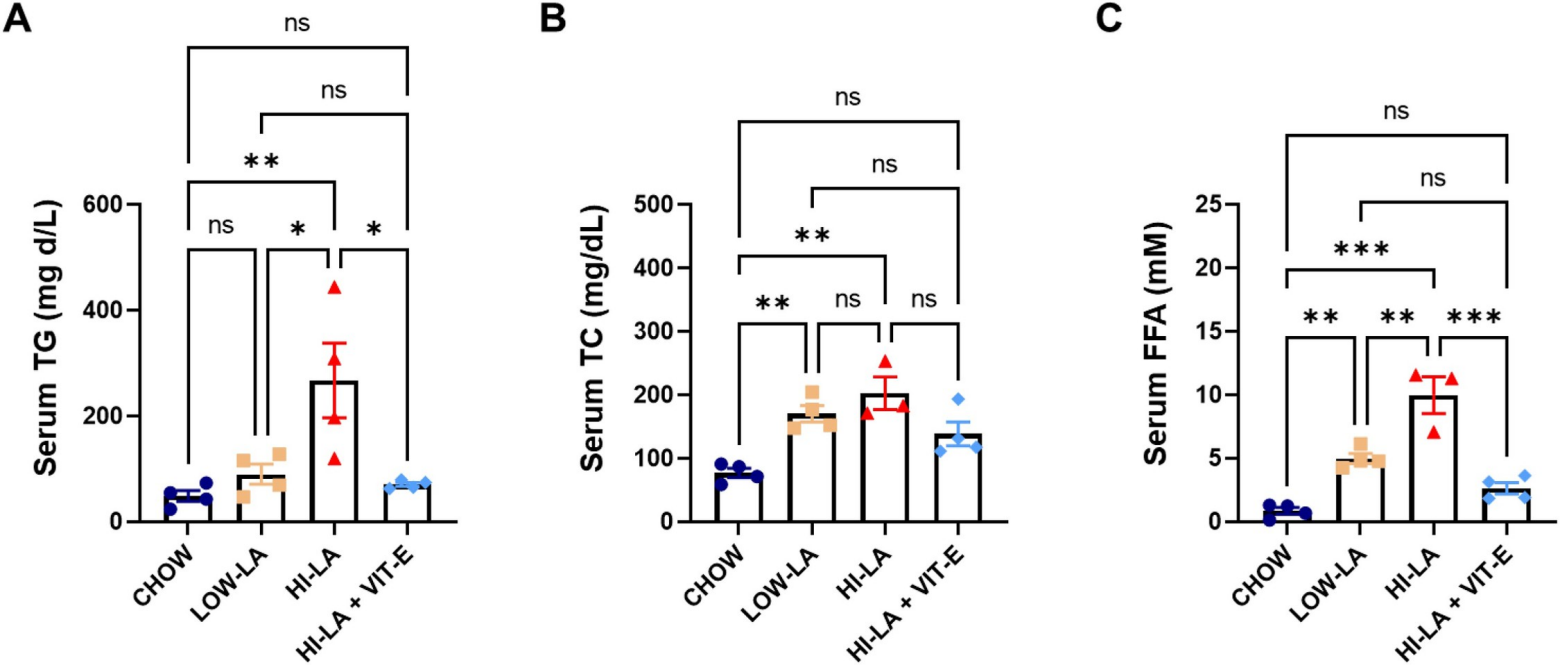
Effects of linoleic acid on serum level of lipids. Serum levels of TG (A), TC (B), and FFA (C) were determined in mice fed with the four types of diets. TG, triglycerides; TC, total cholesterol; FFA, free fatty acids. Data were presented as means ± S.E., *p<0.*05*, **p<0.*01*, ***p<0.*001*, ns, no significance.

Change in adipocyte size and number following the HI-LA diet could result in further adipocyte dysfunction and insulin resistance [21–23]. The impact of HI-LA diet on lipid metabolism was further examined. We found that serum TG level was the highest in the HI-LA diet fed animals, compared to that of the CHOW and LOW-LA groups (Fig 3A). Supplement of VIT-E to HI-LA diet reduced TG level that is comparable to that in the LOW-LA group. Serum TC was also the highest in the HI-LA and significantly different from the CHOW groups, while the HI-LA + VIT-E fed group had only a mild increase in serum TC (Fig 3B). Serum FFA was elevated in both the LOW-LA and HI-LA groups, but the HI-LA group had almost twice as many FFA as that in the LOW-LA group (Fig 3C). Consistently, VIT-E supplementation significantly lowered serum FFA levels to that of the control group.

**Fig 4:**
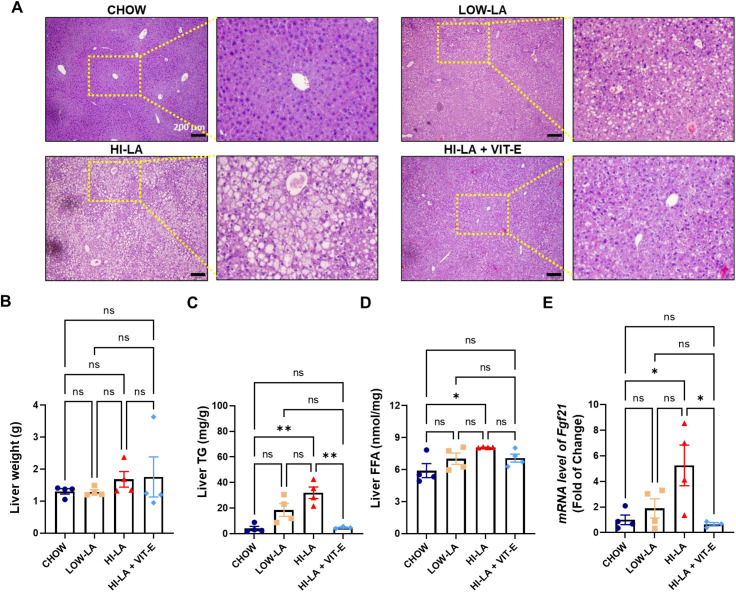
Effects of linoleic acid on hepatic steatosis. Mice were fed with the four types of diets for 20 weeks and the following parameters were determined: representative H&E images of the liver (A), liver weight (B), hepatic levels of TG (C) and FFA (D), and hepatic mRNA level of *Fgf21* (E). Scale bar, 200 μm. Data were presented as means ± S.E., *p<0.*05*, **p<0.*01*, ns, no significance.

Histological examination of the liver sections from the HI-LA diet fed animals, but not those from mice fed with LOW-LA diet or HI-LA plus VIT-E diet, showed severe hepatic steatosis (Fig 4A). In contrast, the liver weight was only mildly increased by HI-LA diet (Fig 4B). The hepatic levels of TG and FFA in the four diet groups mirrored those in the serum with the highest elevation seen in the HI-LA fed mice and the protection of VIT-E against such an elevation (Fig 4C and 4D). These findings were correlated with the gene expression of fibroblast growth factor 21 (*Fgf21*) (Fig 4E), which was known to be increased in response to hepatic steatosis [24]. Not surprisingly, *Fgf21* level was significantly increased in the HI-LA group while only mildly increased in the LOW-LA group. Supplement of VIT-E to HI-LA diet led to the virtually complete silence of this induction, consistent with the dramatic effect of VIT-E on hepatic steatosis and obesity.

**Fig 5:**
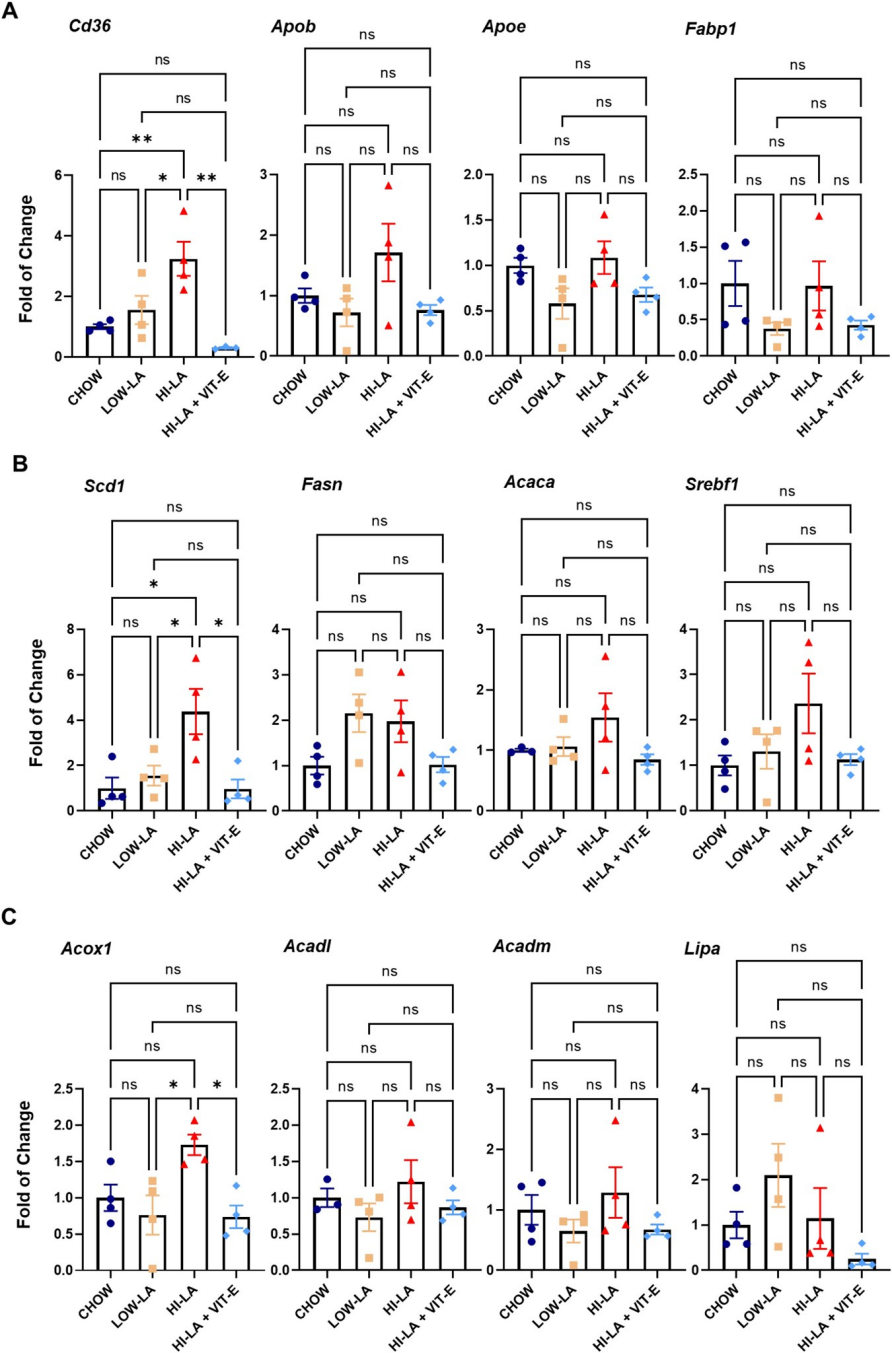
Effects of linoleic acid on expression of genes related to lipid metabolism in the liver. Mice were fed with the four types of diets and the mRNA levels were determined for genes related to lipid transport (A), lipid synthesis (B), and lipid oxidation/hydrolysis (C). Data were presented as means ± S.E., *p<0.*05*, **p<0.*01*, ns, no significance.

Investigating the potential change of multiple other genes involved in lipid metabolism, we observed a significant increase in the expression of cluster of differentiation 36 (*Cd36*), and also a notable but not significant increase of apolipoprotein B-100 (*ApoB*), both of which are involved in lipid transportation, in HI-LA fed animals relative to the other groups (Fig 5A). Expression of stearoyl-CoA desaturase 1 (*Scd1*), acetyl-CoA carboxylase alpha (*Acaca*), and sterol regulatory element-binding transcription factor 1 (*Srebf1*) were upregulated in the HI-LA fed group relatively to the CHOW group, which were reduced to the CHOW level in HI-LA + VIT-E groups (Fig 5B). These genes are involved in anabolic lipid metabolism [25, 26]. In contrast, expression of several genes involved in lipid oxidation did not seem to be altered by the HFD, except fatty acid oxidation enzyme acyl-CoA oxidase 1 (*Acox1*), which was notably higher in the HI-LA fed group than in the other groups (Fig 5C). These findings suggest that HI-LA diet could significantly promote lipid accumulation through lipid transportation and lipid synthesis, which in turn also simulates lipid oxidation to some degree.

### A high linoleic acid diet causes more significant liver damage

**Fig 6:**
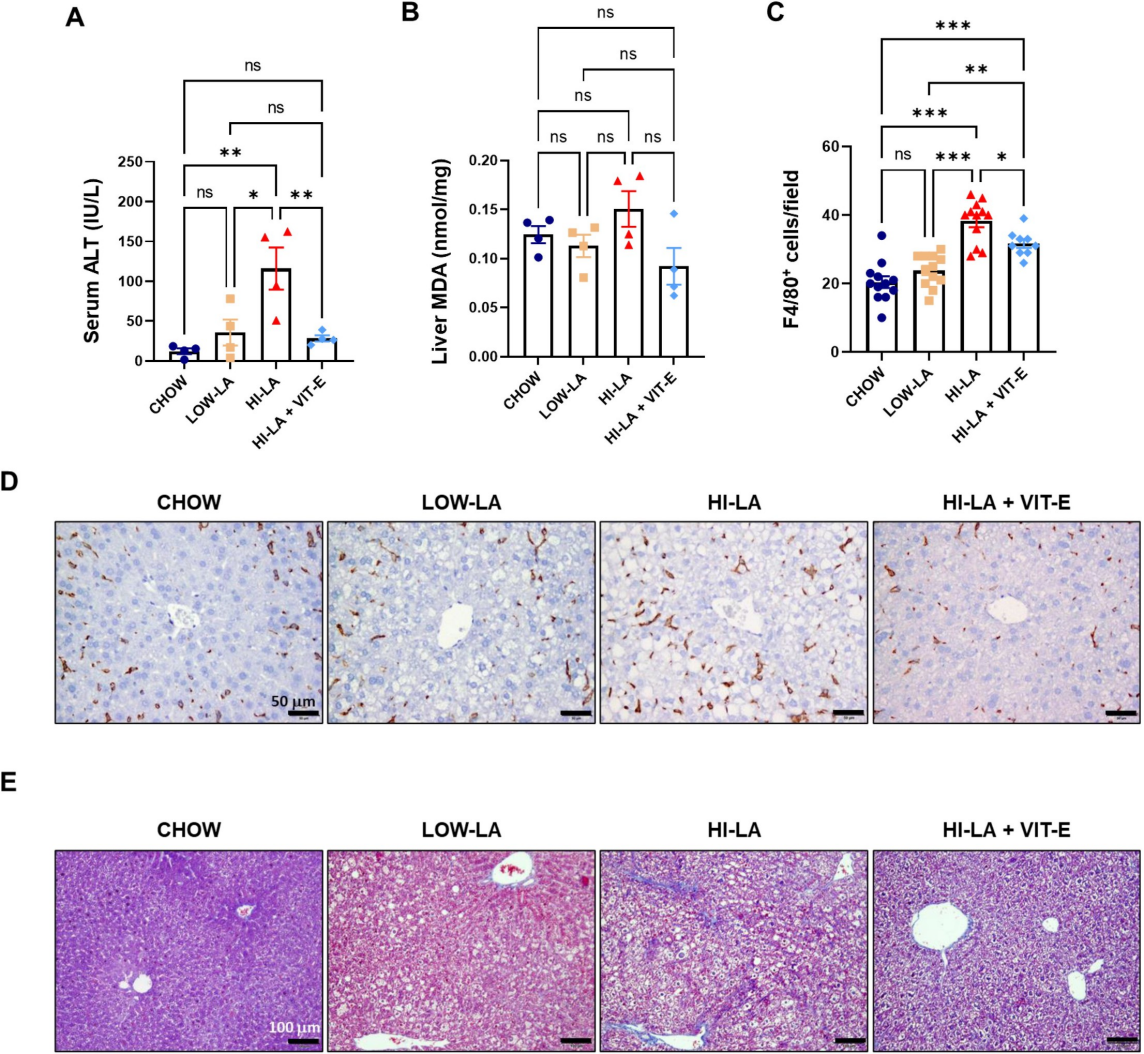
Effects of linoleic acid and vitamin E on liver damage. Mice were fed with the four types of diets and the following parameters were determined: serum level of ALT (A), hepatic level of MDA (B), and number of F4/80-positive cells per field (C, 200x). (D) Representative F4/80 staining images of livers. Scale bar, 50 μm. (E) Representative trichrome staining images of livers. Scale bar, 100 μm. Data were presented as means ± S.E., *p<0.*05*, **p<0.*01*, ***p<0.*001*. ns, no significance.

In addition to altered lipid metabolism, HI-LA diet also caused more severe liver damage as indicated by a much higher level of serum ALT, which was more than tripled, compared to that of the LOW-LA group, which was only mildly increased over the CHOW group (Fig 6A). Increasing the amount of VIT-E in the HI-LA diet significantly protected the mice against liver injury, with the serum ALT level reduced to nearly that of CHOW group (Fig 6A). Interestingly, the level of MDA, which is resulted from the lipid peroxidation of PUFA, only mildly elevated in the HI-LA group, which nevertheless was reduced notably by VIT-E supplement (Fig 6B). On the other hand, HI-LA diet, but not LOW-LA diet, induced significant inflammation, as indicated by the increase of F4/80-positive cells in the liver (Fig 6C and 6D), and notable fibrosis, as indicated by trichrome staining (Fig 6E). Supplement of VIT-E to the HI-LA diet ameliorated the level of hepatic inflammation and fibrosis in mice (Fig 6C–6E). These results suggest VIT-E may protect the liver directly and indirectly through multiple mechanisms.

## Discussion

The present study shows evidence of the role of a high dietary intake of LA in the development of fatty liver disease and several other metabolic abnormalities, and that adequate VIT-E intake that leads to a proper VIT-E/PUFA ratio can ameliorate these effects.

### An increased level of LA contributes significantly to the pathogenic effects of HFD whereas a proper ratio of Vit-E/PUFA can antagonize these effects

Conventional wisdom is that increased energy intake is the primary contributor to obesity and metabolic dysfunction [27]. The present study showed that a high LA composition in a HFD structure can cause a greater weight gain in mice without increasing food uptake, consistent with previous study showing the obesogenic importance of LA [15, 16]. The HI-LA diet promoted subcutaneous adipocyte hypertrophy, which can cause increased lipolysis and greater circulating FFA as observed in the HI-LA fed animals. This increased burden of circulating FFA on the liver may have contributed to the hepatic steatosis and pathogenesis seen in HI-LA fed animals, including changes in the hepatic lipid metabolism. Our studies support the notion that lipids accumulate through enhanced lipid transport and *de novo* synthesis, although lipid oxidation may be increased to some degree in response. Furthermore, our study confirms histopathological evidence of hepatic fibrosis, inflammation, and injury in animals fed a HI-LA diet.

Evidence has shown a beneficial effect of an adequate VIT-E intake to match the increased PUFA intake [28, 29]. A recent review concludes that while not applicable to all diets due to the peroxidability of different PUFAs, the VIT-E to PUFA ratio in the diet should be 0.4 to 0.6 mg *α*-tocopherol per gram of PUFA to maintain nutritional adequacy [29]. This dietary recommendation may need to be re-examined and a higher ratio may be warranted. In the present study, we supplemented VIT-E to the HI-LA diet to increase the ratio of VIT-E to PUFA from 0.9 to 1.9, which is comparable to that in the chow diet and in the LO-LA diet (Table 1). Our results demonstrated that the pathological changes seen in the low VIT-E/PUFA diet structure (HI-LA diet) were reversed by the high VIT-E/PUFA diet structure (HI-LA plus VIT-E). Our observations confirmed the notion that increase vitamin E intake to match the increased PUFA intake can be beneficial.

### There could be multiple mechanisms accounting for the pathogenic effect of linoleic acid and for the beneficial effect of Vitamin E

Many studies have implicated LA in human pathogenesis. For example, one study indicated that the exposure of infants to LA is a potential contributor to childhood obesity [30], and another study found that the obesogenic effect of a Western diet is related to LA’s conversion into several endocannabinoids, which play roles in hyperphagia [31, 32]. The role of PUFA, including LA, in oxidative stress has been implicated in many diseases [33–35]. In the liver system, previous studies have suggested the role of high LA [36, 37], but not SFA [38–40], in the alcoholic liver injury through increased lipid peroxidation.

PUFA is less energy efficient. PUFA oxidation in mitochondria has been shown to produce less energy per unit than SFA or MUFA, and PUFA plays a role in the initiation of torpor in hibernating mammals, an energy conservation adaptation [41, 42]. On the other hand, PUFA is more pathogenic due to its proneness to peroxidation, which, in combination of other mechanisms, can lead to lipotoxicity, cell death and tissue injury [43]. However, in the present study, the level of MDA, a major product of lipid peroxidation, is only mildly increased following HI-LA diet, which, however, was greatly reduced by an increased VIT-E intake. This result may suggest that either the small increase of MDA is sufficient to tip the balance toward the injury, or additional oxidative mechanisms beyond lipid peroxidation could exist, which needs more investigation in the future. LA may also mediate its pathological effect through the conversion into pro-inflammatory mediators [44–46], thus measuring the level of these mediators could be important in HI-LA fed mice.

There are several possible mechanisms to explain the beneficial effect of VIT-E in antagonizing the metabolic changes caused by PUFAs. Firstly, the anti-lipid peroxidation effect from VIT-E may play a role [12]. Like all fat-soluble vitamins, VIT-E is typically concentrated and stored within adipose tissue, where it can inhibit the resident PUFA from lipid peroxidation [47, 48]. Furthermore, VIT-E has effects beyond the termination of lipid peroxidation chain reactions. Secondly, VIT-E also shows anti-inflammatory effects by inhibiting enzymes involved in the conversion of LA into pro-inflammatory mediators [44–46]. These inflammatory mediators have been recognized as key contributors to several metabolic abnormalities, including obesity and liver injury seen here. Indeed, the present study also confirms that VIT-E supplements suppressed the inflammation in HI-LA fed mice.

In summary, the present study has demonstrated the key contribution of a high LA level in liver pathogenesis in a mouse model of NAFLD, and the beneficial effect of increasing VIT-E uptake to antagonize LA. Thus a proper VIT-E/PUFA ratio in a diet could be important to avoid the detrimental effect of PUFA.

## Supporting information

S1 Table Determination of the calorie contribution of major fatty acids in the diet. (PDF)

S2 Table Determination of the level of Vitamin E in the diet and the ratio vs that of PUFA. (PDF)

S1 Checklist The ARRIVE guidelines 2.0: Author checklist. (PDF)
