# Supplementary material for: Ability of high fat diet to induce liver pathology correlates with the level of linoleic acid and Vitamin E in the diet
Source: PLoS One. 2023 Jun 2;18(6):e0286726. doi: 10.1371/journal.pone.0286726 (PMC10237441; doi:10.1371/journal.pone.0286726)
Supplement: S1 Table — (PDF) [file pone.0286726.s001.pdf]

Table S1. Determianation of the carlorie contribution of major fatty acids in the diet

CHOW diet (LabDiet 5053)

	g/kg diet	cal/kg diet	**cal/kg diet**	% CAL	Corrected % Cal
**TOTAL SFA**	7.9	71.1	3606	1.97	2.24
**TOTAL MUFA**	9.7	87.3	3606	2.42	2.75
**Total PUFA**	26.5	238.5	3606	6.61	7.50
PUFA-LA	22.8	205.2	3606	5.69	6.45
Other PUFA	3.7	33.3	3606	0.92	1.05
**% cal (PUFA+MUFA+SFA)**				11.01	12.48
**% cal (total fat)**				12.48	
Correcting factor				1.134	

LOW-LA HFD (Dyet #104946)

**TOTAL SFA**	**SFA/Oil (g/100g)**	**SFA/Oil (g/kg)**	**Oil/Diet (kg/kg)**	**SFA/Diet (g/kg)**	**cal SFA/kg diet**	**cal/kg diet**	**% cal SFA**	**% cal SFA (Corrected)**
Coconut oil	82.5	825	0.108	89.1				
High Oleic Sunflower	9.86	98.6	0.05	4.93				
Flax	8.98	89.8	0.0092	0.82616				
Total				94.85616	853.70544	4247	20.10	21.74

**TOTAL MUFA**	**MUFA/Oil (g/100g)**	**MUFA/Oil (g/kg)**	**Oil/Diet (kg/kg)**	**MUFA/Diet (g/kg)**	**cal MUFA/kg die**	**cal/kg diet**	**% cal MUFA**	**% cal MUFA (Corrected)**
Coconut oil	6.31	63.1	0.108	6.8148				
High Oleic Sunflower	83.7	837	0.05	41.85				
Flax	18.4	184	0.0092	1.6928				
Total				50.3576	453.2184	4247	10.67	11.54

**TOTAL PUFA**	**PUFA/Oil (g/100g)**	**PUFA/Oil (g/kg)**	**Oil/Diet (kg/kg)**	**PUFA/Diet (g/kg)**	**cal PUFA/kg die**	**cal/kg diet**	**% cal PUFA**	**% cal PUFA (Corrected)**
Coconut oil	1.7	11.7	0.108	1.2636				
High Oleic Sunflower	3.8	38	0.05	1.9				
Flax	67.8	678	0.0092	6.2376				
Total				9.4012	84.6108	4247	1.99	2.15
PUFA-LA								1.00
Other PUFA								1.15
**% cal (PUFA+MUFA+SFA)**							32.77	
**% cal (total fat)**							35.43	
Correction factor							1.0813	

HI-LA HFD (Dyet #104947 and Diet #181162)

**TOTAL SFA**	**SFA/Oil (g/100g)**	**SFA/Oil (g/kg)**	**Oil/Diet (kg/kg)**	**SFA/Diet (g/kg)**	**cal SFA/kg diet**	**cal/kg diet**	**% cal SFA**	**% cal SFA (Corrected)**
coconut oil	82.5	825	0.07	57.75				
High oleic sunflower	9.86	98.6	0.042	4.1412				
Flaxseed	8.98	89.8	0.0084	0.75432				
Hi LA safflower	6.2	62	0.043	2.666				
Soybean	14.9	149	0.003	0.447				
Total				65.75852	591.82668	4243.174	13.95	15.06

**TOTAL MUFA**	**MUFA/Oil (g/100g)**	**MUFA/Oil (g/kg)**	**Oil/Diet (kg/kg)**	**MUFA/Diet (g/kg)**	**cal MUFA/kg die**	**cal/kg diet**	**% cal MUFA**	**% cal MUFA (Corrected)**
coconut oil	6.31	63.1	0.07	4.417				
High oleic sunflower	83.7	837	0.042	35.154				
Flaxseed	18.4	184	0.0084	1.5456				
Hi LA safflower	14.4	144	0.043	6.192				
Soybean	22.1	221	0.003	0.663				
Total				47.9716	431.7444	4243.174	10.18	10.99

**TOTAL PUFA**	**PUFA/Oil (g/100g)**	**PUFA/Oil (g/kg)**	**Oil/Diet (kg/kg)**	**PUFA/Diet (g/kg)**	**cal PUFA/kg die**	**cal/kg diet**	**% cal PUFA**	**% cal PUFA (Corrected)**
hydrogenated coconut oil		11.7	0.07	0.819				
High oleic sunflower		38	0.042	1.596				
Flaxseed		678	0.0084	5.6952				
Hi LA safflower		746	0.043	32.078				
Soybean		57.6	0.003	0.1728				
Total				40.361	363.249	4243.174	8.56	9.24
PUFA-LA								8.00
Other PUFA								1.24
**% cal (PUFA+MUFA+SFA)**							32.68	
**% cal (total fat)**							35.29	
Correction factor							1.0797	

Note

1. Levels of different oils or fatty acids are supplied by the manufacturer or obtained from the USDA's food data central (https://fdc.nal.usda.gov/).
2. Total calorie per kilogram of diet is based on the total amount of protein, fat and carbohydrate in each diet as idicated in the manufacturer's speicifications. Each kilogram of oil (fat) generates about 9 calorie of energy.
3. Percentage calorie contribution of linoleic acid (LA) is predefined as 1% for Low-LA high fat diet, and as 8% for HI-LA high fat diet as customorily manufactured. The non-LA PUFA contribution is calculated by deducting LA contribution from the total PUFA conribution.
4. Calarie contribution from the total amount of fat and that of the sum of specific fatty acids can be different. The ratio of the two values are used as an adjusting factor to correct the calorie contribution of individial fatty acids, which are presented here and in Table 1.
