# Supplementary material for: Ability of high fat diet to induce liver pathology correlates with the level of linoleic acid and Vitamin E in the diet
Source: PLoS One. 2023 Jun 2;18(6):e0286726. doi: 10.1371/journal.pone.0286726 (PMC10237441; doi:10.1371/journal.pone.0286726)
Supplement: S2 Table — (PDF) [file pone.0286726.s002.pdf]

Table S2. Determianation of the level of Vitatmin E in the diet and the ratio vs that of PUFA

CHOW diet (LabDiet 5053)				Vit E/Diet (mg/kg)	PUFA/Diet (g/kg)	Vit E/PUFA (mg/g)
99 IU vitamin E/kg (0.45 mg/IU)				44.55	26.5	1.68

LOW-LA HFD (Dyet #104946)	Vit E/Oil (mg/100g)	Vit E/Oil (mg/kg	Oil/Diet (kg/kg)	Vit E/Diet (mg/kg)	PUFA/Diet (g/kg)	Vit E/PUFA (mg/g)
hydrogenated coconut oil	0.11	1.1	0.11	0.12		
High oleic sunflower	41.1	411	0.05	20.55		
Flaxseed	0.47	4.7	0.01	0.04		
Total				20.71	9.40	2.20

HI-LA HFD (Dyet #104947)	Vit E/Oil (mg/100g)	Vit E/Oil (mg/kg	Oil/Diet (kg/kg)	Vit E/Diet (mg/kg)	PUFA/Diet (g/kg)	Vit E/PUFA (mg/g)
hydrogenated coconut oil	0.11	1.1	0.070	0.08		
High oleic sunflower	41.1	411	0.042	17.26		
Flaxseed	0.47	4.7	0.008	0.04		
Hi LA safflower	34.1	341	0.043	14.66		
Soybean	12.2	122	0.003	0.37		
Total				32.41	40.36	0.80

HI-LA HFD + Vit E (Dyet #18116	Vit E/Oil (mg/100g)	Vit E/Oil (mg/kg	Oil/Diet (kg/kg)	Vit E/Diet (mg/kg)	PUFA/Diet (g/kg)	Vit E/PUFA (mg/g)
hydrogenated coconut oil	0.11	1.1	0.07	0.077		
High oleic sunflower	41.1	411	0.042	17.26		
Flaxseed	0.47	4.7	0.008	0.04		
Hi LA safflower	34.1	341	0.043	14.66		
Soybean	12.2	122	0.003	0.37		
Subtotal				32.41		
Vit E in Vitamin Mix	**Vit E/Vit Mix (mg/g)**		**Vit Mix/Diet (g/kg**	**Vit E/Diet (mg/kg)**		
75 IU /10 g Vitamin Mix (0.45 mg/IU)	3.375		13.5	45.5625		
Total				77.97	40.36	1.93

Note

1. Amount of Vit E in chow diet (LabDiet 5053) is provided in the manufacturer's specification.
2. Amount of Vit E in high fat diet without Vit E supplement is calculated based on the level in different oils (USDA's food data central: https://fdc.nal.usda.gov/).
3. Almount of Vit E in the Vitamin Mix (AIN-93VX 310025, Dyets, Inc) is 75 IU/10 g mix (https://dyets.com/vitamin-mix-compositions/).
4. 1 IU of the synthetic form of Vit E is equivalent to 0.45 mg of alpha-tocopherol (https://ods.od.nih.gov/factsheets/VitaminE-HealthProfessional/)
4. Levels of PUFA are calculated in Supplemental Table S1
